# Seamless integration of sewer system topology and tree location data: An algorithm to diagnose the potential impact of tree roots on pipes and propose rearrangement solutions

**DOI:** 10.1016/j.heliyon.2023.e23382

**Published:** 2023-12-07

**Authors:** David Martínez, Sergi Bergillos, Lluís Corominas, Marc Comas-Cufí, Eusebi Calle

**Affiliations:** a Institute of Informatics and Applications, University of Girona, Maria Aurèlia Capmany 61 (Edifici PIV), 17003, Girona, Spain; b Catalan Institute for Water Research, Emily Grahit 101, 17003, Girona, Spain

**Keywords:** Wastewater leakages, Risk assessment, Pipe failures, Underground networks, Environmental protection, Rearrangement solutions

## Abstract

Wastewater networks are subject to several threats leading to wastewater leakages and public health hazards. External elements such as natural factors and human activities are common causes of wastewater leakages and require more in-depth analysis. Prevention and rehabilitation work is essential to secure wastewater networks and avoid pipe failures. This work presents a new algorithm that allows for the seamless integration of sewer topology and tree location data to diagnose the potential impact of tree roots on pipes. The algorithm also proposes tree rearrangement options that balance the cost of tree rearrangement with the cost of pipe repair. The paper also showcases a real-world case study in the city of Girona to evaluate the performance of the presented algorithms for a specific case focusing on tree roots as a natural factor. Results show that it is possible to optimally rearrange a number of the trees with the greatest impact, significantly minimizing pipe failures and wastewater leakages (82% risk reduction with only rearranging a 12% of the most impactful trees). The rearrangement solution not only protects the environment and prevents public health hazards, but also achieves a positive economic payback during the operational period of the pipes, saving up to 1.33M€ for a tree rearrangement of 7%. The presented methodology is applicable to other natural or human factors.

## 1 Introduction

A wastewater network is formed by a conglomeration of underground pipes and maintenance holes that work together in order to collect and drain wastewater from households or industrial centers to wastewater treatment plants. Once treated, the water is returned to the environment or reused for beneficial purposes such as agriculture, irrigation, or even potable water supplies [1], ever more widely accepted by the general public [2]. Hence, wastewater networks are critical infrastructures and essential assets for the proper functioning of society and the economy [3], [4].

The wastewater network is subject to several threats that lead to leakages and, thus, significant economic losses and public health hazards [5]. Pipe failures cause not only direct economic costs through repairs, but also indirect costs such as damage to infrastructures, disruption to business, and production losses [6]. The concept of “urban water security” emerged to address such vulnerabilities leading to a new understanding of the complex dynamics between human and natural systems and can pave the way to extend the scope of risk management [7].

Christodoulou et al. [8] and Obradović [9] agree that natural factors such as tree roots affect pipe failure hazard rates. In particular, they recommend checking pipes in the proximity of trees and evaluating the possibility of tree rearrangement, although a more in-depth analysis is needed. According to their work, the risk of pipe failure incidents concerning tree roots increases over time and can be exacerbated on old or corroded pipes [10]. Sydney Water [11] agrees with this statement, justifying that the roots of trees planted in the wrong place can find their way into wastewater pipes, causing about 80% of all dry weather sewage overflows and seriously affecting public health and the environment. However, there is an active discussion on whether tree roots are able to crack pipes. According to Hartley [12], tree roots may affect pipes in other ways such as joint intrusion or tensile forces. For example, no matter the individual root size, the total volume of the tree roots in a joint could develop a surface big enough to break the pipe collar.

The concept of Tree Protection Zones (TPZs) is widely known in the world of arboriculture, and is defined as the calculated area above and below ground at a given distance from the tree trunk to provide for the protection of the tree's roots and canopy during construction works [13]. Although the TPZ is tied to tree protection, it can be used the other way around. In other words, although TPZ defines an area where construction works can affect tree roots, this area can also be considered the impact zone where tree roots can break into infrastructures such as wastewater pipes. The Australian Standard [14] is the most widely-accepted method for calculating TPZ, although it has led to discussion [15].

Tree species also influence the hazard rate depending on their capacity to build and extend root systems. Ward and Clatterbuck [16] provided a list of slow-growing tolerant trees considered “sewer-safe”, and Sydney Water [11] a list of fast-growing sensitive species. Hence, planting the right species of tree also reduces pipe failure hazard rates [9]. Moreover, Östberg et al. [17] identified which tree species are most likely to crack pipes through fieldwork inspections of wastewater networks. Specifically, it is advisable to conduct CCTV sewer surveys at increased intervals, particularly when previous surveys have revealed a history of high-risk pipe failures [18].

Municipalities often tend to maintain an inventory of trees and their respective species, as well as details about the wastewater network (e.g., pipe age, material, length). Given the extensive datasets available to municipalities, there is potential to apply AI techniques to predict failure risks. Dawood et al. [19] achieve this by performing a literature review on AI algorithms to predict drinking water pipes' risk of failure and highlighted that the main parameters related to this are physical factors such as age, length, diameter, and material. Other factors, including environmental (e.g., tree roots) and operational ones, present a lower effect on pipe failure risk, although they should also be considered.

Apart from the AI approaches, Amiri-Ardakani and Najafzadeh [20] presented a probabilistic framework, considering both natural and human factors, for pipe break rate estimation. Other works require Monte Carlo simulations to predict risk probabilities [21]. Moreover, Vishwakarma and Sinha [22] introduced a prediction method using a fuzzy inference system to assess the comprehensive failure impacts of water pipes based on economic, social, and environmental impacts, operational characteristics, and renewal complexity.

**Figure 1:**
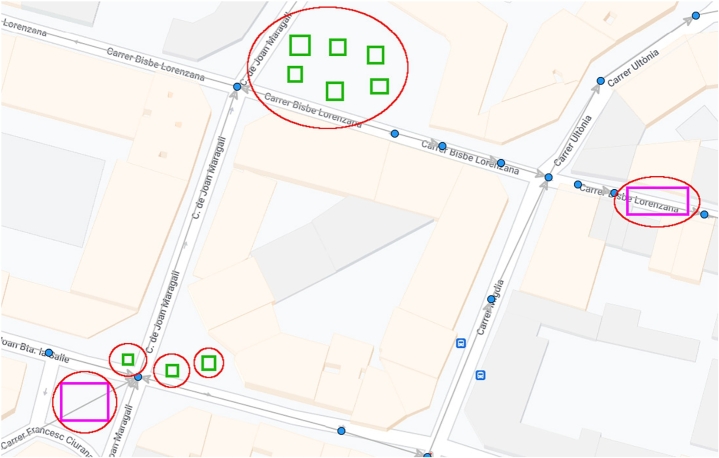
Examples of potential Impact Areas (IAs) drawn in red ellipses considering natural (green rectangles representing urban trees) and human (purple rectangles representing street works) factors in a portion of the wastewater network of Girona (pipes in gray lines, junctions in blue-filled circles).

Human factors such as street works or building constructions have been proven as other of the main factors leading to accidents and also contribute to wastewater leakages and pipe failures [23]. Managing human activities is essential to prevent wastewater leakages. Hence, the concept of TPZ can be applied to other human factors, such as urban construction works, and used as an Impact Area (IA) metric for physical elements that can make their way into wastewater pipes (Fig. 1).

Intelligent tree positioning in cities has recently been applied in other fields of research, such as the strategic planning of trees in a city area to improve the walkability of the outdoor space [24]. However, tree rearrangement has not been studied yet for minimizing the impact of tree roots on pipes and, thus, going towards securing wastewater networks.

Drawing from this background and the availability of relevant databases for pipe and risk element geolocalization, it is possible to automate processes through mathematical algorithms to determine whether it is better to wait and repair pipes when failures or leakages occur or avoid them by rearranging nearby elements. The novelty of this approach is to automatically cross these databases for fast environmental protection and city planning, providing cost-effective impact and risk assessments, as it requires already available data in municipalities and without the need for fieldwork in sewers. The contributions are the following:

1. A new concept of Impact Areas (IAs), which defines the impact zone where physical elements can break into underground network infrastructures, evaluates the impact of the elements and detects the worst threats based on the idea of Tree Protection Zones (TPZs).
2. Mathematical algorithms and methods are introduced to analyze the impact of risk elements on the pipes and evaluate the pipe failure hazard risk on wastewater networks.
3. A novel algorithm provides Element Rearrangement (ER) solutions that minimize pipe failure hazard risk and mitigate wastewater leakages.
4. For a specific case study considering tree roots as natural elements, a comparison between the results obtained and an “all sewer-safe tree city”, which is an ideal city where all trees would be slow-growing or tolerant, quantifies the benefit and wastewater leakage risk reduction of planting “sewer-safe” trees at convenient locations.
5. A method to estimate the probabilities of pipe failures is also introduced and used to extract the expected cost of repair. The original and ER scenarios are then compared and analyzed.
6. The economic savings (i.e., payback) considering the expected cost of repairs and the cost of the ER works are analyzed for both the original and ER scenarios.

The remainder of the paper is organized as follows. Section 2 presents the methodology followed; including an explanation of the general methodology (Section 2.1), the definition of the algorithms and methods (Section 2.2), and the introduction of the case study (Section 2.3). Section 3 illustrate the results and effectiveness and considers the approach described in the paper together with the discussion and future work, including the results of the tree impact and pipe failure risk analyses (Section 3.1), the most damaging trees detection (Section 3.2), the pipe risk reduction from the Element Rearrangement (ER) algorithm (Section 3.3), the pipe failure probabilities and expected cost of repairs (Section 3.4), and the economic savings of the ER algorithm (Section 3.5). Finally, Section 4 summarizes the results and contributions of the paper.

## 2 Materials and methods

The following methods are based on graph theory to build and manage the layer of wastewater pipe networks [25]. Several previous studies have used graph theory in water distribution and wastewater networks: Ahmadullah and Dongshik [26] for designing drinking water networks; Calle et al. [27] for wastewater sensor placement approaches concerning SARS-CoV-2 detection; and Meng et al. [28] for proposing a comprehensive analytical framework for examining the resilience pattern of water distribution systems against topological characteristics (i.e., the correlations between resilience and topological features).

### 2.1 General methodology

**Figure 2:**
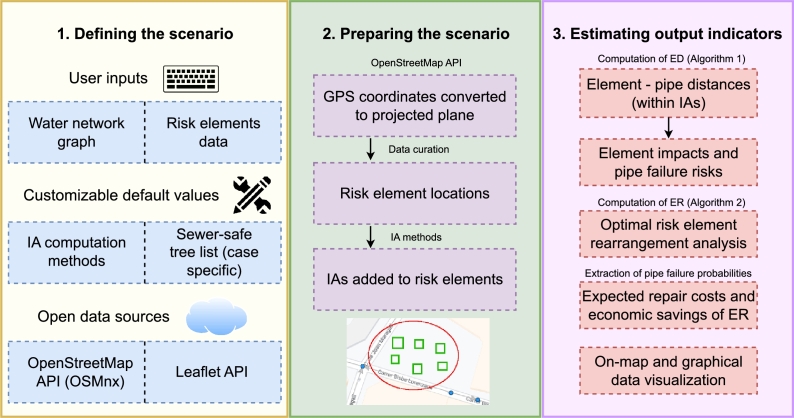
Methodology scheme for the presented methods and algorithms.

The methods and mathematical algorithms presented in this paper involve the following steps: (i) defining the scenario; (ii) preparing the scenario; and (iii) estimating key output indicators (Fig. 2).

#### 2.1.1 Defining the scenario

The required user inputs are (i) a city's water network graph and (ii) the risk elements data. The first parameter (i) must be in a graph format (e.g., Graphml [29]), usually converted from a geographical information format (e.g., GIS) provided by water companies. The second parameter (ii) must include at least the location and, in the case of trees as natural risk elements, an interval (or exact value) of trunk perimeter or diameter and, optionally, the species of the trees. If provided, tree species allow for a more finely-tuned calculation of the Impact Area (IA) depending on the Tree Protection Zone (TPZ) computation method.

A safe radius proportional to the risk element sizes is used to calculate the IAs, which varies in function of the method being considered. In the specific case of trees, Table 1 compares the three most used TPZ methods: Day et al., the Australian Standard, and considering all trees as “sewer-safe”. The last method is used to analyze the gain in pipe failure risks (i.e., failure minimization) in a hypothetical scenario where all the city trees would be “sewer-safe” (i.e., an ideal city where all trees would be slow-growing or tolerant).

**Table 1:** Comparison between the three most used Tree Protection Zone (TPZ) methods used to calculate the Impact Area (IA) in the case of trees as risk elements.

IA	TPZ method	References
Day et al.	6:1 ratio (radius of TPZ:trunk diameter) for tolerant or “sewer-safe” trees and 18:1 for sensitive fast-growing species	[30], [31]
Australian Standard	12:1 ratio (radius of TPZ:trunk diameter) for all trees	[14]
All “sewer-safe”	6:1 ratio (radius of TPZ:trunk diameter) for all trees	[30], [31], [16]

#### 2.1.2 Preparing the scenario

The preparation of the scenario starts with converting the original network coordinates onto a projected EPSG 3857 plane, a Spherical Mercator projection coordinate system popularized by Google Maps and later OpenStreetMap. Next, risk element locations are processed together with their IAs, which depend on the risk element size and type.

In the case of trees as a risk element type, tree diameter is estimated based on trunk perimeter or diameter intervals depending on the original dataset. In the case of diameter intervals, this is considered the upper value (i.e., worst-case scenario). Then, “sewer-safe” trees that are identified through the computation of the Tree Protection Zone (TPZ) may differ based on the method. Finally, the TPZ is computed for each tree based on the selected TPZ method, obtaining the Impact Areas (IAs) and the scenario is ready for the computation of the algorithms.

#### 2.1.3 Estimating output indicators

The impact of a risk element is the value representing the effect of this element on the nearby pipes inside its Impact Area (IA) based on the distances between the element and the pipes. The pipe failure risks are calculated from the aggregation of the affected impacts of each element for each pipe. The calculated IAs are used together with the distances obtained from the execution of the ED algorithm (Algorithm 1) to calculate the element impacts and the pipe failure risks. Furthermore, impacts are used for the optimal analysis of rearrangement to minimize pipe failure risk through the execution of the Element Rearrangement (ER) algorithm (Algorithm 2).

**Algorithm 1:**
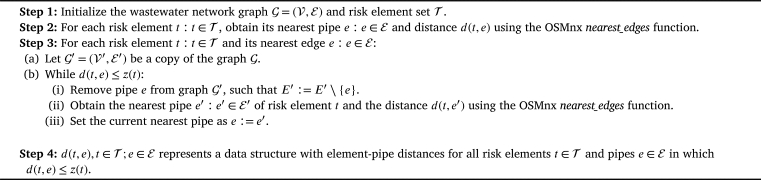
Element - pipe distances (ED) algorithm.

**Algorithm 2:**
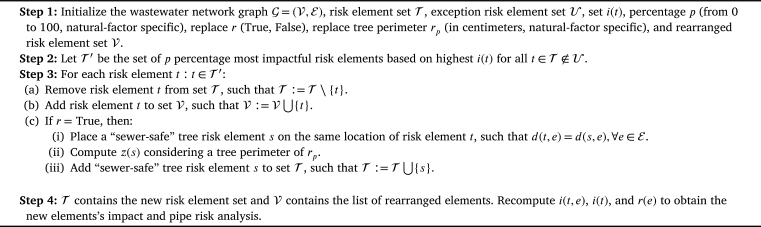
Element Rearrangement (ER) algorithm.

The probabilities of pipe failures are also calculated considering the failure risks, thus enabling the extraction of the expected repair costs. The expected repair costs can be calculated for both the original scenario (i.e., original element locations) and considering rearranged elements (ER algorithm). Furthermore, rich on-map and graphical data visualization of the results are generated to help visualize the numerical results through the open data sources (e.g., data histograms, geographical pipe risk and most-impacting-element maps, line charts, etc.).

### 2.2 Algorithms

In brief, let G=(V,E) be the wastewater network graph, with a *V*-element set of nodes V representing the set of origin (wastewater entries) nodes, the wastewater treatment plant, and junction points, and an *E*-element set of links $E\subset V^{\left|2\right|}$ representing pipes. Additionally, T denotes a *T*-element set of risk elements. Table 2 specifies the notation used for the algorithms.

**Table 2:** Full notation concerning the algorithms.

T	set of risk elements
E	set of wastewater network pipes
z(t),t∈T	Impact Area (IA) of risk element *t*
d(t,e),t∈T;e∈E	distance between risk element *t* and pipe *e*
i(t,e),t∈T;e∈E	impact of risk element *t* on pipe *e*
i(t),t∈T	impact of risk element *t* on the wastewater network
r(e),e∈E	failure risk of pipe *e* based on risk element impact aggregation
p(e),e∈E	probability of failure of pipe *e* (caused by risk elements during its operational period)
l(e),e∈E	length of pipe *e*
c(e),e∈E	expected repair cost of pipe *e* based on *p*(*e*)

First, the element-pipe distances (ED) algorithm (Algorithm 1) makes use of the *nearest_edges* function, a key component of the OSMnx library, as detailed in Boeing's work [32]. The *nearest_edges* function serves a straightforward purpose: it identifies the closest water network pipe *e* to a specific geographic point, representing the location of a risk element *t* within the context of the study.

The ED algorithm plays a pivotal role in determining the set d(t,e), which characterizes the distances between each identified risk element *t* and all the water network pipes *e* that are situated within the risk element *t* Impact Area (IA). For each risk element *t* and its nearest pipe *e*, acquired through the *nearest_edges* function, the ED algorithm systematically checks if the pipe *e* falls within the IA of the risk element *t*. If it does, the algorithm removes this pipe *e* from the original wastewater network G and executes the *nearest_edges* function once more to identify the next nearest pipe $e^{\prime }$. This process continues until the algorithm identifies pipes and element-pipe distances that fall outside the IA for all the risk elements. In essence, the ED algorithm harnesses the capabilities of spatial analysis and geographic data to precisely compute these d(t,e) distances.

**Figure 3:**
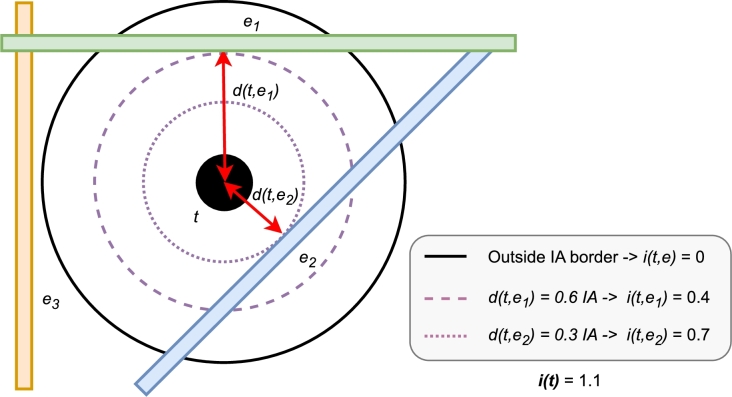
Example of risk element impacts *i*(*t*,*e*) computation within an IA (*t* as a tree).

Then, Equation (1) describes the risk element impacts for each pipe i(t,e),t∈T,e∈E. In other words, the impact of element *t* on a pipe *e* is defined as a normalized value between 0 and 1. The maximum value represents the pipe passing through the center of the IA, and the minimum represents the pipe passing just at the edge of the IA (Fig. 3). If the pipe is outside the IA, the value is considered 0.

(1) $$i(t,e):=\max\{1-\frac{d(t,e)}{z(t)},0\}$$

The impact aggregation of risk element i(t) on the whole wastewater network is simply the summation of risk element *t* impact over the network pipes e∈E (Equation (2)). Note that the impact aggregation i(t) values can be greater than one, and taking the example of Fig. 3, a value of i(t)=1.1 represents the impact aggregation of the pipe $e_{1}$ ($i(t,e_{1})=0.4$) and the pipe $e_{2}$ ($i(t,e_{2})=0.7$), which are those inside the IA of risk element *t*.

(2) i(t):=∑i(t,e),e∈E

The same procedure is followed in Equation (3) to obtain the pipe failure risk of each pipe r(e) considering all risk elements, i.e., the summation of each risk element t∈T impact over the network pipe *e*.

(3) r(e):=∑i(t,e),t∈T

Although the pipe failure risk r(e) is a quantitative measure, Table 3 shows a proposal of qualitative assessment, which assists in interpreting the results of the measure presented later in the results section. Note that the assessment value intervals can be adjusted with more research or using other requirements.

**Table 3:**
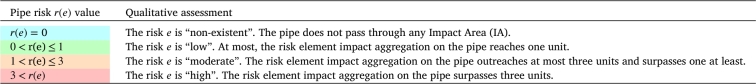
Proposal of pipe qualitative risk assessment.

The Element Rearrangement (ER) algorithm (Algorithm 2) minimizes the pipe risk by rearranging a portion of the most impactful risk elements. The algorithm contemplates two options: (i) risk element removal or (ii) element replacement in the case of trees as a natural risk element type, as trees can be replaced with a smaller “sewer-safe” tree alternative). Moreover, the algorithm also considers an exception element list U for cases where it is not possible to remove specific elements (e.g., in the case of trees for cultural or historical significance or technical challenges; or in the case of street building constructions for the original location being the only available option).

In the context of tree roots as a natural factor, the ER algorithm operates as follows: it begins by selecting the top *p* percentage of the most impactful trees from the entire set of risk elements T, creating a new set denoted as $T^{\prime }$. Subsequently, each tree *t* in $T^{\prime }$ is removed from T and integrated into V, signifying the exclusion of the specific tree *t* from the original set. When the “replace” option *r* is activated, a new “sewer-safe” tree *s* with a perimeter of $r_{p}$ is introduced at the same location as the original tree *t*. The Impact Area (IA) of the new tree *s*, denoted as z(s), is then calculated, and this newly introduced tree *s* is incorporated into the set of risk elements T.

Consequently, the algorithm proceeds to recompute the impact of the trees and the risks associated with the pipes (i.e., i(t,e), i(t), and r(e)). As a result, the T set now contains the updated risk element set, while V maintains a record of the relocated elements.

To extract the probabilities of pipe failures caused by risk elements p(e),e∈E, a new customizable threshold $R_{fail}$ sets the value of risk r(e) in which there is a 100% probability of failure of pipe *e* during its operating time period, often considered 30 years (i.e., if $r(e)=R_{fail}$, then p(e)=1) [33]. Depending on the desired $R_{fail}$ value, some probabilities may be greater than one due to data outliers (i.e., if $R_{fail}<r(e),e\in E$), which may indicate the probability of more than one failure during the operation period. From the $R_{fail}$ threshold, the failure probability of each pipe p(e) can be calculated through its risk value r(e) normalized with $R_{fail}$ (Equation (4)).

(4) $$p(e):=\frac{r(e)}{R_{fail}}$$

According to most sources consulted, including ABM Consulting, entire pipe sections affected by physical elements failures such as tree roots must be replaced. The repair costs depend on multiple factors, such as pipe diameter, material, and terrain. The total cost of pipe repair per meter *R* has to include the material, placement, earthmoving works, and eventually affected services (e.g., economic losses from a temporary road closure), which will depend on the country of the case study.

The expected cost of a failure repair for each pipe c(e),e∈E can be estimated through failure probability p(e) and the concept of expected value (i.e., multiplying the total cost of repair per meter *R* by its length and the likelihood pipe failure will occur p(e)), such that (Equation (5)):

(5) c(e):=R×l(e)×p(e)

Finally, the total expected repair cost for the whole wastewater network *C* is simply the summation of the expected repair cost of each pipe, such that (Equation (6)):

(6) C:=∑c(e),e∈E

### 2.3 Case study

**Figure 4:**
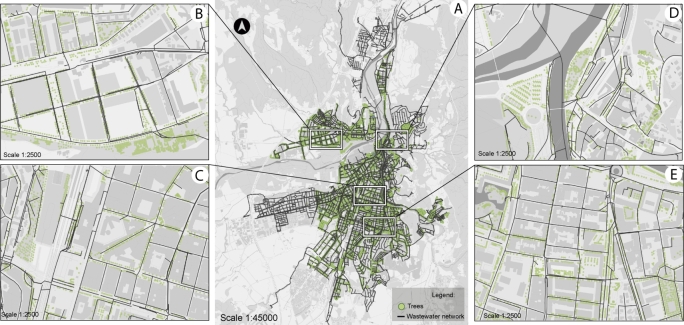
The case study area of Girona, showing tree locations labeled as green points and wastewater network pipes as black lines (QGIS generated). A - Entire city area; Scale 1:45000. B - Zoomed area of Domeny neighborhood; Scale 1:2500. C - Zoomed area of Eixample neighborhood; Scale 1:2500. D - Zoomed area of Barri Vell neighborhood; Scale 1:2500. E - Zoomed area of Montilivi neighborhood; Scale 1:2500.

The usefulness of the methods and algorithms presented in this paper is illustrated in the city of Girona, Catalonia (northeast of the Iberian Peninsula, see Figs. 4, 4A, 4B, 4C, 4D, 4E), considering the entire dataset of the city trees as natural factor risk elements. Girona, with its 102,666 inhabitants and 47,446 households (2.2 citizens per household), is a typical compact Western Mediterranean city [34]. Its urban area extends 12.7 km^2^ on a rivers' crossing, has a population density of 8,139 hab/km^2^, an average slope of 5.1, and an altitude range (difference between the minimum and maximum altitudes) of 177 m. Its sewage network consists of more than 6,000 maintenance holes, resulting in a large network totaling 265 km of pipes. The basic topological characteristics of the network layer are 7,946 nodes (*V*); 8,303 edges (*E*); an average nodal degree of 2.1 ($\bar{D}$); a diameter of 11,071 meters (∅); and an average shortest path length of 47 meters ($\bar{d}$).

On the one hand, the company Cicle de l'Aigua del Ter, which manages the wastewater network of Girona, provided the topological data from the city sewer network in geographic information system (GIS) format files, including feature geometry and attributes. First, the GIS files needed to be converted to a GraphML file format (i.e., to an XML-based format [29]). The GraphML file format is compatible with the Network Robustness Simulator (NRS) [35], used for graph analysis and the execution of the algorithms. The final wastewater network graph is in a unique file in GraphML format containing both nodes and edges and their attributes. Next, data verification and reconciliation processes are performed based on previous research [27].

On the other hand, the city tree data was obtained from the Girona Open Data portal [36], which includes an extended dataset (in CSV format) of 32,881 trees updated in January 2023 with the following attributes: (i) scientific name; (ii) common name in Catalan; (iii) trunk size, as perimeter intervals in centimeters (20-50 / 50-80 / 80-120 / +120); (iv) tree pit frame, as size of the larger side intervals in centimeters (-40 / 40-90 / +90); (v) x coordinate, in UTM ETRS89 format; and (vi) y coordinate, in UTM ETRS89 format. In order to apply the algorithms to this case study, it is necessary to prepare the data of the tree dataset according to our methodology, converting the coordinates and estimating tree diameters.

## 3 Results and discussion

The results are grouped and presented alongside the discussion, and future work is also introduced at the end of each subsection. The results presented in this section are the following: (i) tree impact and pipe failure risk analysis based on the ED algorithm (Algorithm 1) computation; (ii) most impactful tree detection; (iii) pipe risk minimization through the ER algorithm (Algorithm 2); (iv) extraction of pipe failure probabilities caused by tree roots and the expected cost of repairs; and (v) the actual economic savings (i.e., payback) of the ER algorithm. The results have been obtained using an Ubuntu 22.04 LTS server (CPU AMD Ryzen 5 5600X, 32 GB RAM). All the computations have been spawned in a Python [37] notebook (Jupyter Hub).

### 3.1 Tree impact and pipe failure risk analysis

**Figure 5:**
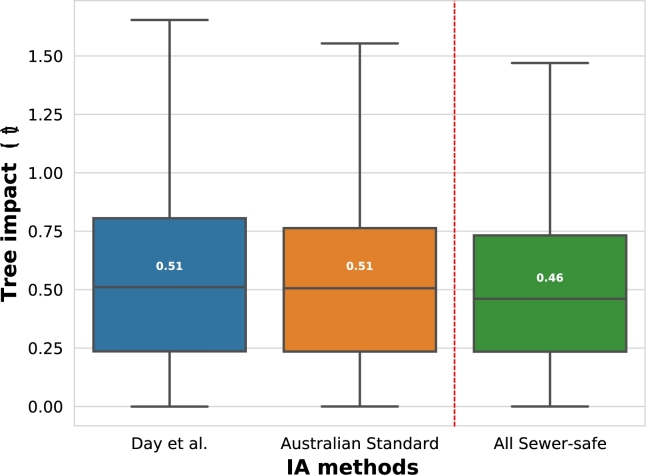
Histogram of tree impacts i(t),t∈T: Day et al. comparison with Australian Standard (AS) IA methods, with the All Sewer-safe method to contrast (without outliers).

Tree impacts i(t) have been calculated based on the element-pipe distances obtained from the ED algorithm (Algorithm 1). Fig. 5 illustrates the histogram comparison of tree impacts i(t)>0 for Day et al. and Australian Standard (AS) TIA methods. It is worth noting that the tree impact data distribution is almost equal for both Day et al. and AS methods (i.e., a median of 0.51), which demonstrates that the AS is an excellent approximation, without the need for tree specie data, for the IA computation. In contrast, Fig. 5 also shows the tree impact i(t) results in a hypothetical situation where all city trees were “sewer-safe” with the same trunk size and location as the actual ones (i.e., identical tree sizes above the surface with much less tree root areas below). The comparison between the Day et al. and AS methods and the All Sewer-safe suggests a significant minimization of pipe failure risk, proving that the median of tree impacts would be reduced by 10% if all the city trees were “sewer-safe”. However, more research and other case studies are needed to check this tendency.

**Figure 6:**
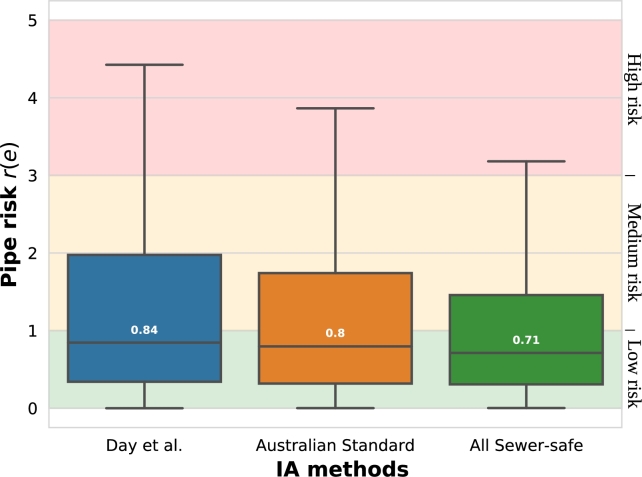
Histogram of pipe risk r(e),e∈E grouped by the three Impact Area (IA) computation methods (without outliers).

The histogram of pipe failure risks concerning tree roots r(e)>0 is shown in Fig. 6, where the background colors represent the qualitative failure risk assessment values (see Table 3). The data distribution follows a similar pattern to tree impacts, with the Day et al. method presenting a slightly higher 0.84 median compared to the 0.8 of the AS. As the AS method keeps showing an excellent approximation, it has been selected as the IA method for the rest of the results. The All Sewer-safe method reveals a more significant reduction in pipe risks data distribution, as low as 0.71, with very little presence of high-risk pipes.

The potential impact analysis of tree roots on pipes in wastewater networks throughout multi-layer crossing was a gap in the current literature. It is worth noting that the pipe failure risk r(e) is a relevant quantitative measure that will be very useful for planning and prioritizing preventive actions on wastewater network pipes.

Future work will be conducted to consider pipe material in the computation of failure risks which, in practice, affects the failure hazard rate [19]. In addition, it would be worthwhile to analyze the effect pipe depth has on risk element impacts. Finally, it would also be interesting to verify our approach with the actual city failure records, as this is, to the best of our knowledge, the first study that quantifies pipe failure risk concerning tree roots at a theoretical level. Our approach does not require sewer fieldwork and is based only on the topology of the network and the tree inventory. The obtained data can also be used to improve the existing Artificial Intelligence (AI) algorithm predictions.

### 3.2 Most impactful tree detection

Table 4 shows the top 10 most impactful trees considering the Australian Standard IA method, illustrating that a small percentage of trees cause the most impact on pipes due to the large number of upper-bound outliers on the data (as shown in Fig. 5) and the significant 44% decrease in the values between the first and the tenth-placed tree. The most impactful trees are expected to be large or in a critical location where many pipes are present (e.g., street crossroads), or both. The majority of the most impactful trees have large IAs, except for number seven, the *Pinus pinea*. A manual check on the location of this tree revealed that it is placed in a critical spot, on a roundabout with a union of seven pipes. It is also worth noting that the most present species in the top 10 are the Celtis australis and the Tilia platyphyllos, which are not considered “sewer-safe” and are well-known for their relatively extensive root systems.

**Table 4:** Top 10 of the most impactful trees in Girona (IA method: Australian Standard).

No.	Scientific name	Trunk per. (cm)	Trunk diam. max. (cm)	IA A.S. (m)	Tree impact i(t)
1	Celtis australis	80-120	38.20	4.60	4.98
2	Tilia platyphyllos	80-120	38.20	4.59	4.59
3	Magnolia grandiflora	50-80	25.46	3.06	4.35
4	Melia azedarach	80-120	38.20	4.58	4.19
5	Platanus x hispanica	>120	50.93	6.11	3.77
6	Celtis australis	80-120	38.20	4.58	3.67
7	Pinus pinea	20-50	15.92	1.91	2.86
8	Celtis australis	>120	50.93	6.11	2.86
9	Tilia platyphyllos	>120	50.93	6.11	2.78
10	Tilia platyphyllos	>120	50.93	6.11	2.77

**Figure 7:**
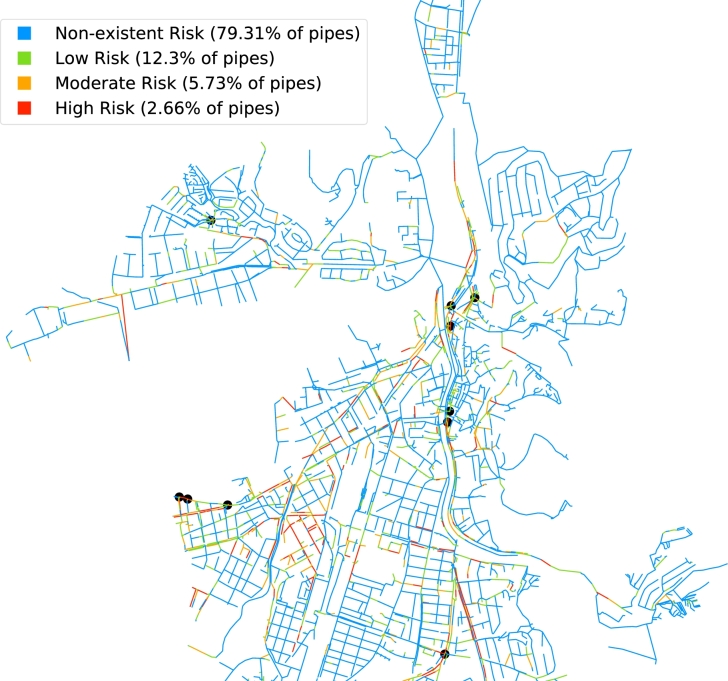
Pipe risk and top 10 most impactful trees (black dots) in part of the wastewater network of Girona.

Fig. 7 visualizes the central part of the wastewater network of Girona represented with plane coordinates, with the color of each pipe representing the risk category and the black dots representing each location of the top 10 most impactful trees. The algorithms generate high-quality PDF maps as well as interactive HTML maps (available on the dedicated public repository [38]), both generated automatically, clearly helping to identify critical pipe risk areas and tree locations. As can be seen in the figure, the location of the most impactful trees matches, in most of the cases, where pipe failure risks are high or moderate, showing in a visual way that these trees are significantly affecting the pipe failure risks.

The most impactful trees and the pipe failure risk map are extremely useful reports for city councils and wastewater network managers, providing an excellent first image of the current scenario. They can be generated easily and without the necessity for additional resources, lots of data, or fieldwork in sewers, in contrast with the existing literature [17], [18]. After this preliminary assessment, decision-makers may require the application of additional methods to minimize the risks, such as localized CCTV inspections in the most critical areas.

In line with the advances in smart cities, future research may develop intelligent tree-planting approaches to indicate in which city zones trees can be planted without being a risk to the wastewater network. In any case, a prioritized list of the most impactful trees concerning wastewater pipes is highly useful information for cities to plan future proceedings.

### 3.3 Element Rearrangement (ER): pipe risk reduction algorithm

**Figure 8:**
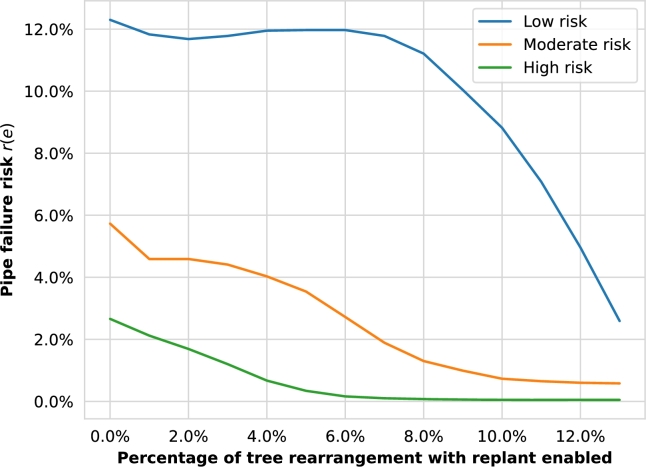
Element Rearrangement (ER) algorithm pipe failure risk minimization grouped by risk categories (Girona tree dataset as a risk element, Australian Standard method, with replant enabled).

The Element Rearrangement (ER) algorithm is expected to decrease the pipe risk significantly by rearranging a portion of the most impactful trees obtained from the previous analysis. Fig. 8 proves this statement showing the Element Rearrangement (ER) algorithm risk reduction considering the Australian Standard TPZ method with tree replacement enabled from 0 to 13% of the dataset trees (i.e., within the percentages that present a clear improvement). The replacement approach considers planting a “sewer-safe” alternative tree in the same spot as the original one, with an assumed trunk perimeter of 50 cm (i.e., the upper value of the smallest trunk perimeter interval of the case-study dataset). With a rearrangement of only 4% of the dataset trees, the number of high-risk pipes is reduced drastically by 75%, and medium-risk ones by 30%. Moreover, the number of medium-risk pipes is reduced significantly by 77% with a ER of 8%. Finally, the number of low-risk pipes is also reduced sharply by 79% with a ER of 13%. With a small percentage of rearranged trees, the pipe failure risk can be lowered substantially, especially for high-risk pipes.

**Figure 9:**
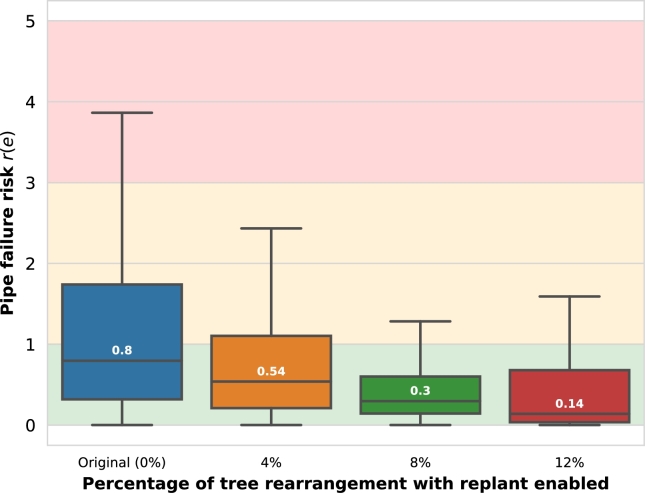
Aggregated pipe failure risk minimization (Element Rearrangement (ER) algorithm, Australian Standard method, with replant enabled, without outliers).

Fig. 9 shows the aggregated pipe failure risk r(e) of the previously categorized risks through the application of the ER algorithm (Australian Standard method, with replant enabled). The risk median is reduced steadily by 33% (4% ER), 62% (8% ER), and 82% (12% ER) from the original tree dataset. For higher ER percentages, the reduction of pipe failure risks tends to stabilize.

The ER algorithm results show a clear benefit of the rearrangement of the city's most impactful trees in terms of pipe failure risks, thus preventing environmental and public health hazards by avoiding wastewater leakages caused by tree roots.

For future research, it would be interesting to consider an automatic feature in the algorithms that would detect if a replanted “sewer-safe” tree does not reduce the risk significantly compared to the high-risk tree it replaced. In this case, a warning should appear along with a recommendation not to plant a tree in that location.

### 3.4 Pipe failure probabilities and expected cost of repairs

The probabilities of pipe failures caused by tree roots have been calculated for the case study for both the original tree dataset and the rearranged tree scenarios. Based on the proposal of the pipe risk qualitative assessment shown in Table 3, a value of $R_{fail}=5$ has been proposed and introduced in the case study. This decision was taken considering that the risk value of r(e)>3 is high and the interval of the medium risk qualitative category is two units (i.e., from 1 to 3). Therefore, a risk of r(e)>=5 may be considered extreme and is only present in a few data outliers in this case study. An $R_{fail}$ of the maximum value of r(e),e∈E is a poor approach as a few extreme data outliers r(e)>10 would significantly affect the whole sample and consider extremely low probabilities on low- and medium-risk pipes.

The expected cost of the pipe failure repairs caused by tree roots has been calculated based on the original scenario, and the cost of pipe repairs of €230 per meter (*R*= 230). According to ABM Consulting, this estimation is valid in Spanish case studies when considering the material to be new 300 mm diameter PVC pipes. The expected cost resulted in about €5.14 m, which is reasonable as the repair works are considered during the entire operating lifetime of the pipes (approx. 30 years), as mentioned in the methods section.

### 3.5 Economic savings of the Element Rearrangement (ER) algorithm

**Figure 10:**
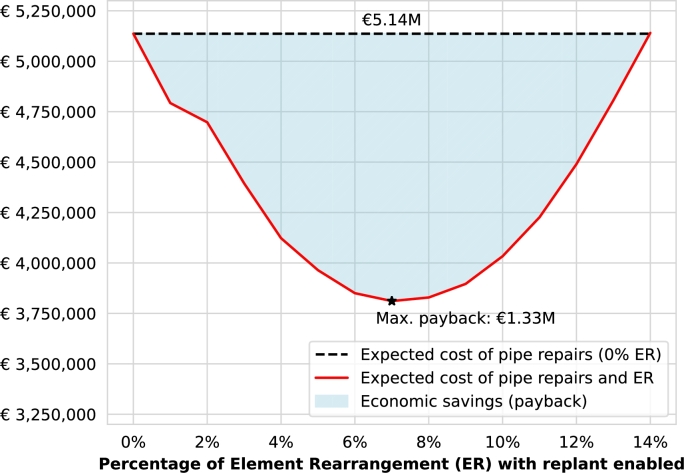
Economic savings of the Element Rearrangement (ER) approach.

The actual economic savings of the Element Rearrangement (ER) approach with tree replant enabled are calculated based on the expected reduction in repair costs from the original scenario combined with an estimation of the ER costs (i.e., payback based on the savings from avoiding pipe failures caused by tree roots during the operational period of the pipes). An ER cost of €1,020 is estimated for each tree in Spain based on the following quotes from several local companies: (i) €350 for big tree removals; (ii) €200 to deposit 5 tonnes wood; (iii) €110 for two-hour rental of a dump truck with loading crane; (iv) €340 for removing the tree stump; and (v) €20 for the cost of a new tree. Table 5 summarizes the expected cost of pipe repairs, costs of the ER, and payback from 1 to 13% of ER. The payback increases within the first 7% of ER up to €1.33 m, although it starts decreasing from 8 to 13% of ER as shown in Fig. 10. After this point, the ER costs start to cause economic losses.

**Table 5:** Expected cost of pipe repairs, costs of the Element Rearrangement (ER), and payback from 1 to 13 % of ER.

ER (%)	ECoR (€)	CoER (€)	P (€)
0	5.14 m	– –	– –
1	4.46 m	336k	346k
2	4.03 m	671k	439k
3	3.39 m	1.01 m	742k
4	2.78 m	1.34 m	1.01 m
5	2.29 m	1.68 m	1.17 m
6	1.84 m	2.01 m	1.29 m
7	1.46 m	2.35 m	1.33 m
8	1.15 m	2.68 m	1.31 m
9	878k	3.02 m	1.24 m
10	680k	3.35 m	1.10 m
11	537k	3.69 m	910k
12	465k	4.02 m	646k
13	446k	4.36 m	330k

ER (%) – percentage of Element Rearrangement (ER), ECoR (€) – expected cost of repairs, CoER (€) – cost of ER, P (€) – payback (i.e., economic savings of ER).

The line chart in Fig. 10 visually illustrates the economic savings presented in Table 5. The dashed black line represents the expected costs without rearrangement (i.e., 0% ER), contrasted with the red line representing the expected costs of pipe repairs and ER. The expected cost of pipe repairs and ER red line forms a quadratic function shaping a parabola with the vertex being the maximum payback of €1.33 m for 7% of ER. Despite the initial investment required, the ER algorithm not only prevents environmental and public health hazards by avoiding wastewater leakages caused by tree roots, but also demonstrates a significant economic payback during the pipes' operation lifetime. In the cases where it is prioritized the minimization of pipe risks instead of the maximization of the economic payback, an ER of 14% should be considered.

It would be interesting to extend these results with more case studies once more cities provide open data with detailed information about tree localization and trunk sizes. Fortunately, cities trend to issue increasingly more open data [39] as novel cost-effective methods are emerging to establish city-wide tree inventories [40].

### 3.6 Final thoughts

It is worth noting that the approach in this paper provides innovative methods that can be applied to any city in the world, as the only information needed is wastewater network and risk element data, including their location. The methodology and algorithms can be extrapolated to other water and underground (e.g., telecommunication or electricity) networks. Furthermore, the Impact Area (IA) concept can be applied to not only trees but also any other risk elements, such as street works or building constructions, in order to quantify their impacts and help create secure wastewater and other underground networks. All algorithm definitions, implementations, and output indicator results for the case study, including numerical, on-map, and graphical data visualizations, are available from a dedicated public repository [38].

The algorithms and methods described in this paper provide a simple and cost-effective approach to diagnose the impact of external elements (e.g., natural factors and human activities) on wastewater networks. Our approach uses the Impact Areas, but not actual impact volumes. We believe, that using Impact Areas makes it simple to understand by decision makers. Adding impact volumes implies adding sources of uncertainty, as limited knowledge exists on the volumes of tree roots of different ages and species. Future work will be dedicated to enhance the algorithm by including impact volumes.

## 4 Conclusions

This paper demonstrates that it is possible to perform an automatic diagnosis of potential impacts of tree roots on wastewater pipes, and to propose cost-effective rearrangement options. The Element Rearrangement (ER) algorithm not only prevents environmental and public health hazards, but also obtains a positive economic payback during the operational period of the pipes within the optimal rearrangement percentages. The proposed novel algorithms could also be applied to other natural and human factors. Furthermore, pipe failure probabilities are calculated and used to estimate the expected cost of pipe repairs during their operational period.

For the case study of Girona, the Australian Standard Tree Protection Zone (TPZ) method is the most practical approach to calculate the Impact Areas (IA) of the city trees, showing a tree impact median of 0.51 and pipe risk median of 0.84. The top 10 most impactful trees cause the majority of pipe damage, given the significant difference (44%) within the values for the first and tenth-placed trees. The Element Rearrangement (ER) algorithm reduced the pipe failure risk median considerably (from 0.8 to 0.14) with a small percentage of ER (from 1 to 12% of the trees). Based on the computed pipe failure probabilities, the expected cost of the pipe repairs caused by tree roots is about €5.14 m during the operational period, which is reduced to almost half (€2.78 m) with only a 4% ER. Finally, the economic savings of the ER algorithm show a payback of up to €1.33 m for a 7% rearrangement despite the required initial inversion.

This study illustrates a cost-effective approach for evaluating the influence of external factors on wastewater networks and pipe failure risks, all without the need for fieldwork in sewers. Despite some limitations, the method's utility lies in its global applicability using existing data in municipalities. It serves as a valuable preliminary study for prioritizing preventive measures and providing a detailed initial assessment, making it particularly useful for city councils and wastewater network managers.

## Ethics statement

Review and/or approval by an ethics committee and informed consent were not needed for this study because it solely relies on publicly available data from open and unrestricted sources. The data used in this research contains no personally identifiable information and does not involve human participants or patients. Therefore, all aspects of this study adhere to ethical standards pertaining to the use of publicly accessible data without requiring formal ethical review or informed consent.

## CRediT authorship contribution statement

**David Martínez:** Writing – review & editing, Writing – original draft, Visualization, Validation, Supervision, Software, Project administration, Methodology, Investigation, Formal analysis, Data curation, Conceptualization. **Sergi Bergillos:** Writing – review & editing, Visualization, Methodology, Formal analysis, Data curation, Conceptualization. **Lluís Corominas:** Writing – review & editing, Visualization, Resources. **Marc Comas-Cufí:** Supervision, Conceptualization. **Eusebi Calle:** Writing – review & editing, Visualization, Supervision, Conceptualization.

## Declaration of Competing Interest

The authors declare that they have no known competing financial interests or personal relationships that could have appeared to influence the work reported in this paper.

## Data Availability

The data, algorithms, and code implementations that support the findings of this study are openly available in “Mitigating Wastewater Leakages for Enhanced Network Protection: Risk Assessment and Practical Solutions.” at https://doi.org/10.5281/zenodo.7704666, reference [38].
